# The Effect of Anti-Amoebic Agents and Ce6-PDT on *Acanthamoeba castellanii* Trophozoites and Cysts, In Vitro

**DOI:** 10.1167/tvst.9.12.29

**Published:** 2020-11-23

**Authors:** Lei Shi, Vithusan Muthukumar, Tanja Stachon, Lorenz Latta, Mohamed Ibrahem Elhawy, Gubesh Gunaratnam, Erika Orosz, Berthold Seitz, Albrecht F. Kiderlen, Markus Bischoff, Nóra Szentmáry

**Affiliations:** 1 Dr. Rolf M. Schwiete Center for Limbal Stem Cell and Aniridia Research, Saarland University, Homburg/Saar, Germany; 2 Department of Ophthalmology, The First Affiliated Hospital of USTC, Division of Life Science and Medicine, University of Science and Technology of China, Hefei, People's Republic of China; 3 Institute for Medical Microbiology and Hygiene, Saarland University, Homburg/Saar, Germany; 4 Department of Parasitology, National Public Health Center, Budapest, Hungary; 5 Department of Ophthalmology, Saarland University Medical Center, Homburg/Saar, Germany; 6 Mycology and Parasitology Unit FG16, Robert Koch Institute, Berlin, Germany; 7 Department of Ophthalmology, Semmelweis University, Budapest, Hungary

**Keywords:** *Acanthamoeba*, drugs, cytotoxicity assay, trypan blue assay, non-nutrient agar *E. coli* plate

## Abstract

**Purpose:**

The purpose of this study was to analyze the concentration-dependent effects of biguanides (polyhexamethylene biguanide [PHMB], chlorhexidine [CH]); diamidines (hexamidine-diisethionate [HD], propamidine-isethionate [PD], dibromopropamidine-diisethionate [DD]); natamycin (NM); miltefosine (MF); povidone iodine (PVPI), and chlorin e6 PDT on *Acanthamoeba* trophozoites and cysts, in vitro.

**Methods:**

Strain 1BU was cultured in peptone-yeast extract-glucose medium. Trophozoites or cysts were cultured in PYG medium containing each agent at 100%, 50%, and 25% of maximum concentration for 2 hours. The percentage of dead trophozoites was determined using a non-radioactive cytotoxicity assay and trypan blue staining. Treated cysts were also maintained on non-nutrient agar *Escherichia coli* (*E.* *coli*) plates and observed for 3 weeks.

**Results:**

All tested drugs displayed significant cytotoxic effects on 1BU cells based on the biochemical and staining-based viability assays tested. On non-nutrient agar *E. coli* plates, neither trophozoites nor freshly formed cysts were observed after PHMB, PD, NM, and PVPI treatment, respectively, within 3 weeks. However, CH-, HD-, DD-, and MF-treated cysts could excyst, multiply, and encyst again.

**Conclusions:**

The off-label drugs PHMB, PD, NM, and PVPI are under in vitro conditions more effective against strain 1BU than CH, HD, DD, and MF. Our findings also suggest that the non-nutrient agar *E.* *coli* plate assay should be considered as method of choice for the in vitro analysis of the treatment efficacy of anti-amoebic agents.

**Translational Relevance:**

Ophthalmologists may optimize the treatment regime against *Acanthamoeba* keratitis by pre-testing the in vitro susceptibilities of the *Acanthamoeba* strain against drugs of interest with the non-nutrient *E.* *coli* agar plate assay.

## Introduction

*Acanthamoeba* are free-living, ubiquitous protozoa that can been isolated from fresh water, sea water, swimming pools, bottled water, and even air. *Acanthamoeba* exist in two life forms, active trophozoites and dormant cysts. Trophozoites can infect humans. Encystment occurs as a result of pH changes or adverse changes in oxygen or food supply. Cysts are smaller, round, double walled, and more resistant to stressful and extreme environments. They may be dormant but viable for several years.^1^^–^^3^

In immunocompromised patients, *Acanthamoeba* infection may attack the central nervous system,^4^ whereas immunocompetent subjects may develop *Acanthamoeba* keratitis (AK).^5^ AK incidence is increasing worldwide,^6^^,^^7^ particularly in contact lens wearers. Cosmetic contact lenses have shown even higher risk of infection.^8^ In 2009, an outbreak of AK in the United States was associated with the use of a contact lens solution.^9^ In Germany, with about 80 million inhabitants, only 150 new cases have been reported in a 10-year period.^10^

AK is a serious, sight-threatening disease for which patients need long-term treatment. To date, however, no standardized treatment is available. All clinical use of anti-amoebic drugs is off-label. In addition, to the best of our knowledge, up to now there is no kill-on-contact drug available against *Achantamoeba* spp.

Current drugs used as off-label treatment against AK involve five action modes: (1) membrane-acting agents, such as polyhexamethylene biguanide (PHMB) and chlorhexidine (CH); (2) intracellular targeting agents such as benzalkonium chloride; (3) nucleic acid-acting drugs, such as propadienes and hexamidines; (4) protein synthesis-inhibiting drugs, such as neomycin; (5) enzyme-acting agents, such as miltefosine (MF).^11^ However, there are no completed randomized, controlled, clinical studies to date analyzing the efficacy of these treatment options.

In AK, the most commonly applied eye drops include diamidines and biguanides. The frequently used 0.1% diamidines are propamidine-isethionate (Brolene, PD), hexamidine-diisethionate (Hexacyl, HD), and dibromopropamidine (Golden Eye, DD).^12^^–^^14^ As biguanides, polyhexamethylene-biguanide (polyhexanid) (Lavasept, PHMB), and chlorhexidine (Curasept, CH) are commonly applied.^15^ At the Department of Ophthalmology of Saarland University, the antibiotic eye drop neomycin is regularly used to prevent bacterial superinfection,^16^ and to reduce the bacterial load as a food source for *Acanthamoeba* cells.^15^ Antifungal agents have also been suggested as topical AK treatment option.^17^^,^^18^ Steroid eye drops were suggested as adjuvant in AK cases involving severe inflammation, but should not be used without additional topical antiseptics.^19^^,^^20^ Povidone-iodine and miltefosine were also reported to show anti-amoebic effects, however, in vivo efficacies of these drugs have not been demonstrated yet.^21^^,^^22^

There is still no standardized method to determine the viability of *Acanthamoeba* trophozoites and cysts following anti-amoebic treatment. In the literature, the trypan blue assay is the most commonly described assay. Nevertheless, the lactate dehydrogenase (LDH) assay and the non-nutrient agar *Escherichia coli* (*E. coli*) plate assay also give us important information on trophozoite and cyst viability following in vitro medical treatment.^23^ LDH is released during tissue damage and the released LDH amount refers to the viability of the cells. The trypan blue assay monitors the integrity of cell membranes. The non-nutrient agar *E.* *coli* plate assay is a culture-based assay, showing surviving *Acanthamoeba* growing on an agar plate.

The purpose of this study was to optimize the clinical treatment of AK by analyzing the concentration-dependent effects of the diamidines hexamidine-diisethionate (HD), propamidine-isethionate (PD), and dibromopropamidine-diisethionate (DD); the biguanides PHMB, CH, natamycin (NM), MF, povidone iodine (PVPI); and chlorin e6 photodynamic therapy (PDT) on *Acanthamoeba castellanii* trophozoites and cysts in vitro.

## Materials and Methods

### Medium and Non-Nutrient Agar Preparation

Peptone-yeast-glucose (PYG) medium: 20 g of protease-peptone (Sigma-Aldrich, St. Louis, MO), 1 g of yeast extract (Sigma-Aldrich), 8 mL of a 0.05 M CaCl_2_ (Gruessing GmbH, Filsum, Germany) solution, 10 mL of 0.4 M MgSO_4_•7H_2_O (Gruessing GmbH) solution, 10 mL of 0.25 M Na_2_HPO_4_•7H2O (Sigma-Aldrich), 10 mL of 0.25 M KH_2_PO_4_ (Gruessing GmbH) solution, 1 g of Na Citrate•2H_2_O (Gruessing GmbH), and 10 mL of 0.005 M Fe(NH4)2(SO4)2•6H2O (Carl Roth GmbH, Karlsruhe, Germany) solution were added to 0.95 L of distilled water. The pH of the medium was adjusted to 6.5, and the solution was subsequently autoclaved at 121°C for 15 minutes. Next, 18 g of glucose (Sigma-Aldrich) dissolved in 50 mL of distilled water was filter sterilized and added to the above solution.

Neff's constant-pH encystment medium: 7.46 g of KCL (Gruessing GmbH), 1.97 g of MgSO_4_•7H_2_O (Gruessing GmbH), 44.40 mg of CaCL_2_•2H_2_O (Gruessing GmbH), 84.01 mg of NaHCO_3_ (Gruessing GmbH), and 2.42 g of Tris-HCL (Gruessing GmbH) were dissolved in 1 L of distilled water. The pH of the solution was adjusted to 9.0, and the medium autoclaved at 121°C for 15 minutes.

Page's amoeba saline (PAS): 0.14 g of Na_2_HPO_4_ (Sigma-Aldrich) and 0.14 g of KH_2_PO_4_ (Sigma-Aldrich) were dissolved in 500 mL of distilled water to make solution A; 0.12 g of NaCl (Gruessing GmbH), 4 mg of MgSO_4_•7H_2_O (Gruessing GmbH), and 4 mg of CaCl_2_•2H_2_O (Gruessing GmbH) were dissolved in 500 mL of distilled water to make solution B. Both solutions were autoclaved at 121°C for 15 minutes. As they cooled to room temperature, solutions A and B were mixed.

Non-nutrient agar: 15 g of agar (Sigma-Aldrich) was mixed with 100 mL of PAS and 900 mL of distilled water, then autoclaved at 121°C for 15 minutes.

### Acanthamoeba Isolate

We received the *Acanthamoeba castellanii* strain 1BU from the Unit for Mycotic, Parasitic, and Mycobacterial Infections (FG16) of the Robert Koch Institute, Berlin, Germany. This strain was isolated from a patient with AK.

### Acanthamoeba Cultures

The 1BU trophozoites were grown in tissue culture flasks containing 5 mL of PYG broth medium at 30°C in an airtight container. Encystment was induced by replacing the PYG broth medium with Neff's constant-pH encystment medium after the trophozoites had reached confluence.

Following incubation of the 1BU trophozoites in encystment medium for 1 week at 30°C, cysts were harvested and washed three times with phosphate-buffered saline (PBS; Sigma-Aldrich) by centrifugation (800 × *g*), then resuspended in 5 mL PAS at a concentration of 3.30 × 10^6^ cysts/mL and stored at 4°C until use.

### Anti-Amoebic Agents and Their Preparation

For the experiments, the anti-amoebic agents PHMB (Pharmacy of Saarland Medical University, Homburg/Saar, Germany), CH (Sigma-Aldrich, USA), DD (European Pharmacopoeia, Strasbourg, France), HD (European Pharmacopoeia, Strasbourg, France), PD (Brolene, Patheon UK Ltd., Swindon, UK), natamycin (5% natamycin ophthalmic suspension preserved with 0.02% benzalkonium chloride; Alcon Laboratories, Fort Worth, TX), PVPI (B. Braun, Melsungen, Germany), MF (Sigma-Aldrich, Switzerland), and chlorin e6 (Ce6, ORPEGEN Pharma, Heidelberg, Germany) were used.

We received HD, DD, CH, MF, and Ce6 in powder form, PHMB as a 20% solution, and PVPI as a 7.5% solution. Propamidine isethionate was available as 0.1% Brolene eye drops, and natamycin in 5% Natamet eye-drop form. These agents were dissolved or diluted in PYG medium for treatment of 1BU trophozoites and in PBS for treatment of 1BU cysts (the final concentrations were the respective clinically used concentrations and 50% and 25% of the clinically used concentrations; Table). Trophozoites cultured in PYG medium and cysts cultured in PBS were used as controls.

**Table.** Anti-Amoebic Agent Concentrations in Our Experiments

Agent	Maximum	50% of Maximum	25% of Maximum
PHMB	0.02% (1079 µM)	0.01% (539.5 µM)	0.005% (269.75 µM)
CH	0.02% (396 µM)	0.01% (198 µM)	0.005% (99 µM)
HD	0.1% (1648 µM)	0.05% (824 µM)	0.025% (412 µM)
PD	0.1% (1771 µM)	0.05% (885.5 µM)	0.025% (442.75 µM)
DD	0.1% (1384 µM)	0.05% (692 µM)	0.025% (346 µM)
NM	5% (75106 µM)	2.5% (37553 µM)	1.25% (18776.5 µM)
MF	0.0065% (160 µM)	0.00325% (80 µM)	0.001625% (40 µM)
PVPI	1% (27401 µM)	0.5% (13700.5 µM)	0.25% (6850.25 µM)
Ce6	0.0152% (256 µM)	0.0076% (128 µM)	0.0038% (64 µM)

In LDH and trypan blue assays the three different concentrations below were used. For the non-nutrient *E.* *coli* agar plate assay, the maximum concentration alone was used.

### Photodynamic Therapy using Chlorin e6 (Ce6)

We received chlorin e6 stock solution (Orpegen Pharma, Heidelberg, Germany) in PBS (30 mM). For the experiments, fresh Ce6 was dissolved in PYG medium or PBS at 64 μM, 128 μM, and 256 μM.

Trophozoites were seeded in 96-well plates at 1 × 10^4^/well and allowed to grow for 24 hours in 100 µL PYG medium before photodynamic treatment. After 24 hours, the trophozoites had attached to the bottoms of the plates and reached confluence. The medium was then replaced with PYG medium containing one of three different concentrations of Ce6 (see Methods). A culture with fresh PYG medium lacking Ce6 was used as a negative control. The cultures were incubated at 37°C for 60 minutes in the dark. Next, the cultures were washed with 100 µL PBS 3 times to remove the Ce6-containing PYG medium, and 100 µL fresh PYG medium was added.

Cysts were seeded in 96-well plates, at 2 × 10^4^/well. PBS containing one of three different concentrations of Ce6 (see Methods) was added to each plate. A culture with PBS lacking Ce6 was used as a negative control. The cultures were incubated at 37°C for 60 minutes in the dark. The plates were then centrifuged at 400 × *g* for 5 minutes and washed with 100 µL PBS 3 times to remove the Ce6-containing PBS, which was finally replaced with 100 µL fresh PBS.

Next, we exposed the cultures (trophozoites or cysts) to red (670 nm) light for 13 minutes (24 J/cm^2^). As a negative control without illumination, we kept the cultures after replacement of the Ce6-containing PYG or PBS medium in the dark for the illumination time of the other cultures. The illumination box was developed previously through the Department of Physics of the University of Kaiserslautern (Kaiserslautern, Germany).^24^^–^^26^

### Lactate Dehydrogenase Assay (Cytotoxicity), Trophozoites

The CytoTox 96 Non-Radioactive kit (Promega Corporation, Madison, WI) was used to quantitate LDH activity.

To determinate the cytotoxic effect of anti-amoebic drugs on *A. castellanii* trophozoites, 1 × 10^4^ 1BU trophozoites were incubated in 96-well plates with 100 µL PYG medium/well at 30°C for 24 hours. During this time, the 1BU trophozoites attached to the bottoms of the plates and reached confluence. After 24 hours, PYG medium was replaced by 100 µL of the anti-amoebic agent-containing medium (preparation described above). Negative controls received 100 µL fresh PYG medium, and positive controls were incubated with 100 µL fresh PYG supplemented with 10 µL lysis solution (provided by the kit). Blank controls were incubated with medium or anti-amoebic agent-containing medium, but without trophozoites. All experimental and control plates were incubated for 2 hours at 30°C. As the lysis buffer in this kit cannot lyse cysts, they were not analyzed using this assay.^23^

Next, the 96-well plates were centrifuged at 200 × *g* for 5 minutes, and 50 µL of the supernatant was removed from each well and transferred into a well of a fresh 96-well plate. Then, 50 µL of substrate reagent was added to each well, the solution was carefully mixed, and it was then incubated for 30 minutes at room temperature. The reaction was stopped by adding 50 µL stop solution to each well. The product of the LDH-catalyzed formazan formation was determined spectroscopically with a multimode microplate reader (PerkinElmer Ensight, Waltham, MA) at 490 nm that was blanked with medium or anti-amoebic agent-containing medium, as appropriate. Cytotoxicity (%) was determined as follows:

$$\begin{array}{ccc} & & \mathrm{Cytotoxicity}\;\left(\%\right)=100\times (\mathrm{Experimental}\;\mathrm{LDH}\\ & & \mathrm{Release}OD_{490}-\mathrm{Blank}\;\mathrm{control}\;OD_{490})/\\ & & (\mathrm{Maximum}\;\mathrm{LDH}\;\mathrm{Release}\;OD_{490}\\ & & -\mathrm{Blank}\;\mathrm{control}\;OD_{490}) \end{array}$$

These experiments were repeated five times on different days.

As the dark brown color of PVPI is similar to the color of the LDH reaction, the effect of PVPI on *A. Castellanii* could not be tested using this assay.

### Trypan Blue Assay, Trophozoites, and Cysts

The 1BU trophozoites or cysts were suspended at 2 × 10^4^/100 µL of PYG or anti-amoebic agent-containing medium (see Methods) in 1.5 mL reaction tubes (Sarstedt, Nümbrecht, Germany) and incubated in a Thermomixer (Eppendorf AG, Hamburg, Germany) with a shaking rate of 650 rpm at 30°C for 2 hours. All reaction tubes were then centrifuged at 200 × *g* for 5 minutes, and 80 µL supernatant was removed from each tube. Next, 20 µL of a 0.4% trypan blue solution (Sigma-Aldrich) was added to each tube and the trophozoites or cysts were incubated at room temperature for 5 minutes. The 1BU trophozoites and cysts without trypan blue staining served as negative controls. Next, 10 µL of the trypan blue-treated cells were pipetted onto a hemocytometer (C-Chip, NanoEnTek, Waltham, MA) and bright field images were taken with a Leica DMI4000 B microscope (Leica Microsystems, Wetzlar, Germany) at 10-fold magnification, using Leica Application Software (LAS) version 3.7. These experiments were repeated three times on different days.

Because natamycin formed a suspension due to its high molecular weight, trophozoites or cysts could not be distinguished from natamycin itself; therefore, this assay was not performed on natamycin-treated samples.

### Non-Nutrient Agar *Escherichia Coli* Plate Assay, Cysts

The non-nutrient agar *E.* *coli* plate assay was carried out essentially as described by Narasimhan et al.^27^ and Kowalski et al.^28^

First, *E. coli* strain IM08B17 was grown overnight on sheep-blood agar plates (Becton Dickinson, Heidelberg, Germany) at 35°C. Fresh-grown IM08B colonies were picked with a cotton swab and suspended in PAS (equivalent to a 4.5 McFarland turbidity standard). This suspension was pipetted onto the surface of non-nutrient agar plates and spread carefully using an L-shaped spreader.

Next, 2 × 10^4^ cysts were mixed with 100 µL PBS in the absence and presence of one of the anti-amoebic agents (0.02% PHMB, 0.02% CH, 0.1% HD, 0.1% PD, 0.1% DD, 5% NM, 0.0065% MF, or 1% PVPI), and were incubated at 30°C for 2 hours in a Thermomixer with a shaking rate of 650 rpm. As a negative control, cysts were incubated in 100 µL PBS (without anti-amoebic agents) for 2 hours. As a positive control, cysts were incubated in lysoform (Rossmann GmbH, Berlin, Germany) for 2 hours. Thereafter, 10 µL of each cell suspension was transferred into a fresh tube, mixed with 10 µL of trypan blue solution (0.4%), and incubated for 5 minutes at room temperature. PBS (980 µL) was subsequently added to each tube (diluting the drugs 100-fold) and the cells were carefully suspended by pipetting gently up and down. A 6 x 6 mm^2^ square and crosslines were drawn on the bottom of each non-nutrient agar *E. coli* plate to define the central and peripheral regions, and 10 µL of the diluted trypan blue stained cell suspensions (approximately 20 cysts) were pipetted into the central regions of the plates, and the suspensions were allowed to aspirate for around 10 minutes. The *A. castellanii*-inoculated plates were incubated upside up for 24 hours at 30°C, after which they were sealed with parafilm (Pechiney, Menasha, WI) and incubated upside down for up to 3 weeks at 30°C.

Bright-field images (25 per each central region and 8 for each peripheral region) were taken with a Leica DMI4000 B microscope immediately after the suspensions were desiccated (0 hours), and every 7 days up to 3 weeks, using a 10-fold magnification. These experiments (with each anti-amoebic agent) were repeated four times on independent days.

### Statistical Analysis

Statistical analysis was performed using GraphPad Prism 7.02, by 1-way or 2-way ANOVA tests, followed by Dunnett's or Sidak's multiple comparisons tests. *P* ≤ 0.05 was considered statistically significant.

A 1-way ANOVA test was used to compare groups treated with different anti-amoebic agents, followed by Dunnett's multiple comparison test to compare each drug group (three different concentrations for each drug) with the controls. For Ce6-PDT, a 2-way ANOVA test was used to compare groups with and without illumination, followed by Didak's multiple comparisons test to compare the groups treated with the three different respective concentrations of drug.

The impact of drug treatment on the growth behavior on non-nutrient agar *E. coli* plates was analyzed by 1-way ANOVA followed by Dunnett's multiple comparison test in reference to the control.

## Results

### LDH Assay

**Figure 1.**
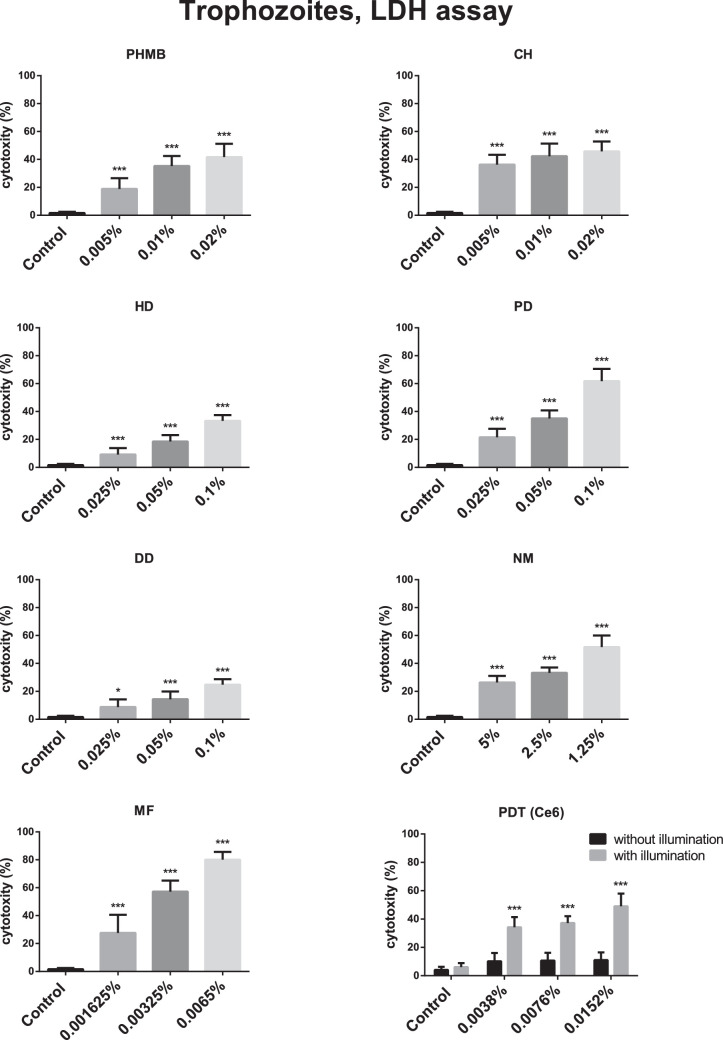
Cytotoxic effect (mean ± standard error) of polyhexamethylene biguanide (PHMB), chlorhexidine (CH), hexamidine-diisethionate (HD), propamidine-isethionate (PD), dibromopropamidine-diisethionate (DD), natamycin (NM), miltefosine (MF), and chlorin e6 photodynamic therapy (PDT) on 1BU trophozoites (LDH assay; n = 5). PHMB, CH, HD, PD, DD, NM, MF, and chlorin e6-PDT increased LDH activity significantly in trophozoite cultures (*P* < 0.01) compared with controls. The use of Ce6 without illumination did not change LDH activity (*P* = 0.96) compared with Ce6 with illumination. * *P* ≤ 0.05; ** *P* ≤ 0.011; *** *P* ≤ 0.005.

Results of the LDH assay for trophozoites (*n* = 5) are displayed in Figure 1.

PHMB, CH, HD, PD, DD, NM, MF, and chlorin e6-PDT increased LDH activity significantly in trophozoite cultures (*P* < 0.01) compared with controls.

The use of Ce6 without illumination did not change LDH activity (*P* = 0.96), compared with Ce6 plus illumination. There was no LDH activity difference between groups treated with different concentrations of Ce6, but without illumination (*P* = 0.09). Illumination alone did not increase LDH activity significantly (*P* = 0.96) compared with controls.

### Trypan Blue Assay

**Figure 2.**
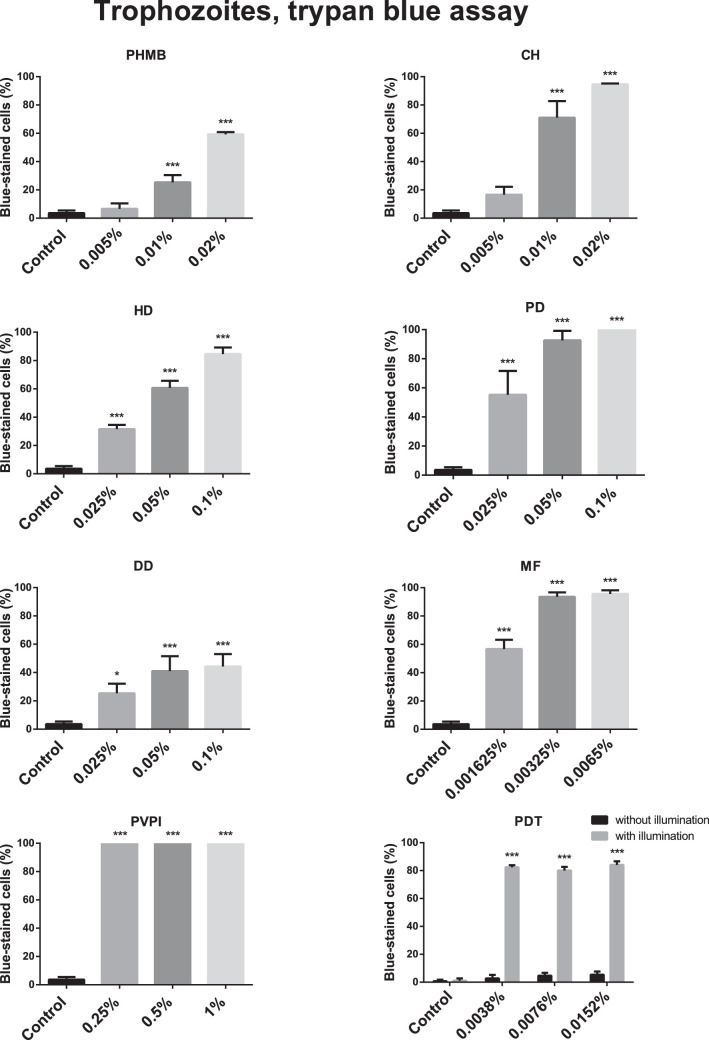
Cytotoxic effect (mean ± standard error) of polyhexamethylene biguanide (PHMB), chlorhexidine (CH), hexamidine-diisethionate (HD), propamidine-isethionate (PD), dibromopropamidine-diisethionate (DD), miltefosine (MF), povidone iodine (PVPI), and chlorin e6 photodynamic therapy (PDT) on 1BU trophozoites (trypan blue assay; *n* = 3). All tested drugs increased the percentage of trypan blue-stained 1BU trophozoites significantly (*P* < 0.01) compared with controls. Concerning different drug concentrations, all displayed significantly (*P* ≤ 0.02) increased cytotoxicity on 1BU trophozoites compared with controls, except 0.005% PHMB (*P* = 0.59), 0.005% CH (*P* = 0.10), and Ce6 without illumination (*P* = 0.99). * *P* ≤ 0.05; ** *P* ≤ 0.011; *** *P* ≤ 0.005.

**Figure 3.**
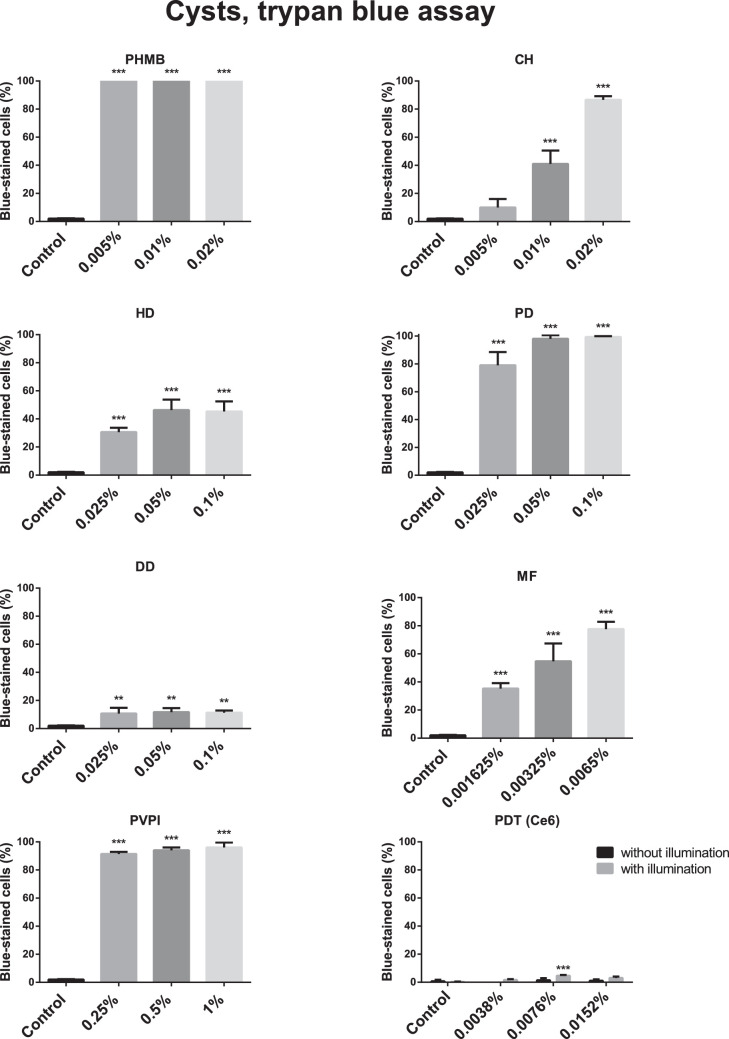
Cytotoxic effect (mean ± standard error) of polyhexamethylene biguanide (PHMB), chlorhexidine (CH), hexamidine-diisethionate (HD), propamidine-isethionate (PD), dibromopropamidine-diisethionate (DD), miltefosine (MF), povidone iodine (PVPI), and chlorin e6 photodynamic therapy (PDT) on 1BU cysts (trypan blue assay; *n* = 3). All tested drugs increased the percentage of trypan blue-stained 1BU cysts significantly (*P* < 0.01) compared with controls. All drug concentrations displayed significantly (*P* < 0.01) increased cytotoxicity to 1BU cysts compared with controls, except 0.005% CH (*P* = 0.29), Ce6 without illumination (*P* = 0.94), and 0.00095% or 0.0038% Ce6 with illumination (*P* = 0.14; *P* = 0.06). * *P* ≤ 0.05; ** *P* ≤ 0.011; *** *P* ≤ 0.005.

The cytotoxicity of the different agents as assessed by use of the trypan blue assay is shown in Figures 2 and 3.

All the tested drugs increased the percentage of trypan blue-stained 1BU *trophozoites* significantly (*P* < 0.01) compared with controls. Every tested drug concentration displayed significantly increased cytotoxicity to 1BU trophozoites compared with the controls (*P* < 0.01), except 0.005% PHMB (*P* = 0.59), 0.005% CH (*P* = 0.10), and Ce6 without illumination (*P* = 0.99). Illumination alone (*P* = 0.99) or the use of different Ce6 concentrations without illumination (*P* = 0.09) did not increase trypan blue positivity compared with the controls.

All tested drugs increased the percentage of trypan blue-stained 1BU cysts significantly (*P* < 0.01) compared with the controls. Every tested drug concentration displayed significantly increased cytotoxicity to 1BU cysts compared with controls (*P* < 0.01), except 0.005% CH (*P* = 0.29), Ce6 without illumination (*P* = 0.94), 0.00095% or 0.0038% Ce6 with illumination (*P* = 0.14; and *P* = 0.06), and illumination alone (*P* = 0.94).

### Non-Nutrient Agar *E.* *Coli* Plate Assay

**Figure 4.**
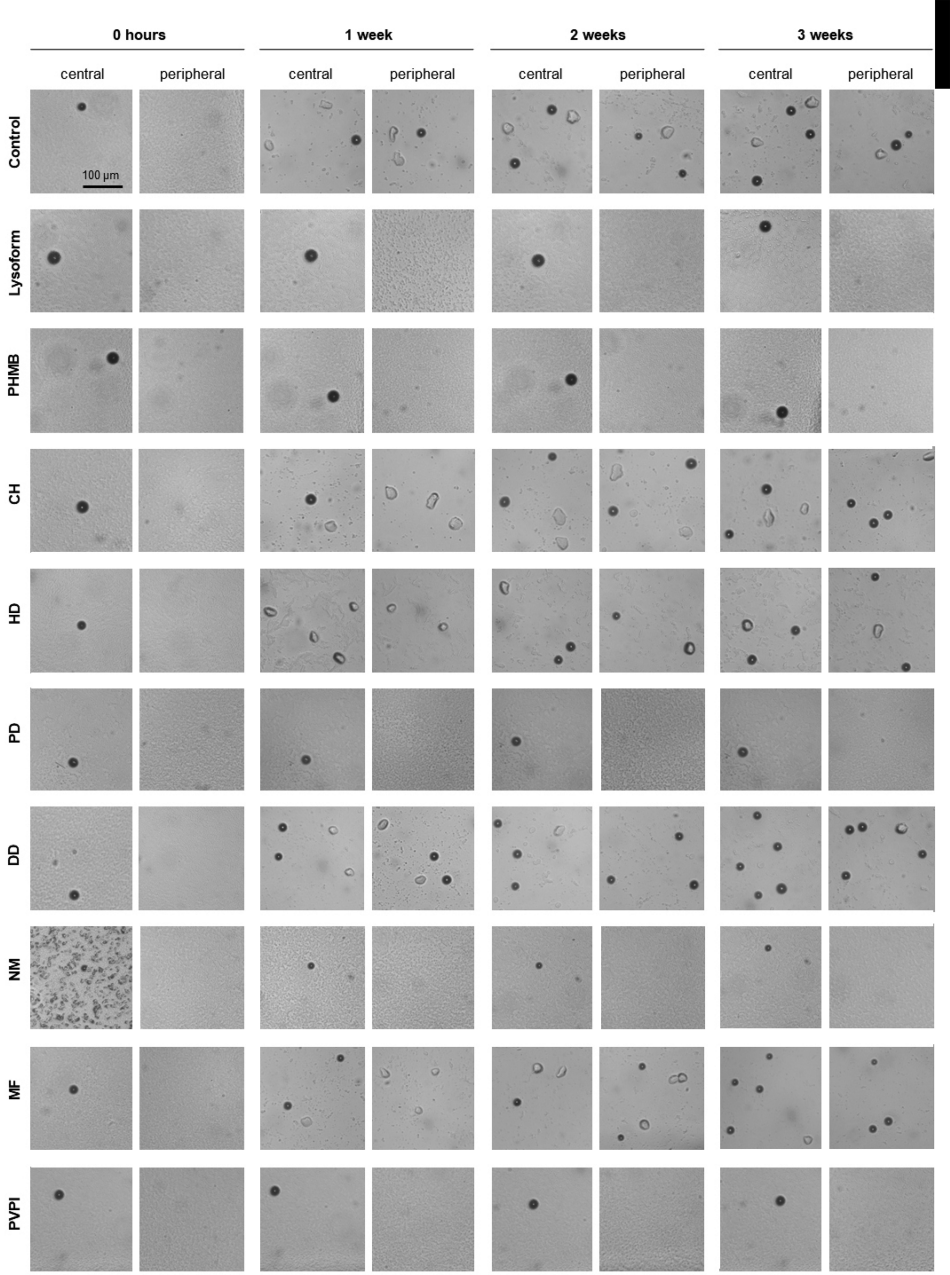
Impact of the drug treatment on the encystment and growth behavior of *A. castellani* 1BU cysts on non-nutrient agar *E. coli* plates. Cysts were incubated in PBS medium either in absence of drugs (control) or with the drugs and concentrations indicated (0.02% PHMB, 0.02% CH, 0.1% HD, 0.1% PD, 0.1% DD, 5% NM, 0.0065% MF, or 1% PVPI), subsequently stained with trypan blue and afterward diluted in medium to a cell density of 2.000 cysts/mL. There were 10 µl of the solution (approximately 20 cysts) were pipetted onto the centers of fresh non-nutrient agar *E. coli* plates. Cysts containing solutions were allowed to aspirate for 10 minutes before the first set of images were recorded by bright field microscopy. Cyst-inoculated plates were subsequently incubated at 30°C for up to 3 weeks and images of the central and peripheral regions were taken at the time points indicated. Images are representative of four independent biological experiments. The disinfectant lysoform served as killing control.

Images of the non-nutrient agar *E. coli* plates at the center and periphery of each plate at four different time points are displayed in Figure 4. On all plates, remnants of the original cysts remained observable at the same place during the entire follow-up, irrespective of whether they excysted or not. In the lysoform-treated control group, no trophozoites or fresh cysts emerged during follow-up.

In the control groups, the CH, HD, DD, and MF-treated groups, fresh, normally shaped trophozoites were visible at 1 week after treatment, and the trophozoites could move out from the center to the peripheral area of the plates. In these groups, encystment happened again after 1 week.

In the PHMB, PD, and PVPI-challenged groups, neither trophozoites nor freshly formed cysts could be observed at all time points monitored.

When placing the NM-treated cysts suspensions onto the non-nutrient agar *E. coli* plates, NM crystals were also deposited on the central regions of the plates, irrespective of the dilution, and formed aggregates on the surface. These structures, however, disappeared along with time, and no trophozoites and freshly formed cysts were observed on any of the plates inoculated with the NM-challenged cysts suspensions.

**Figure 5.**
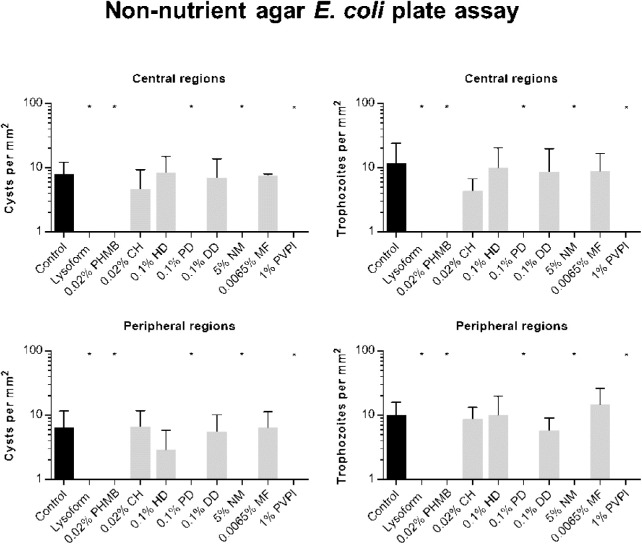
Trophozoite/cyst formation of drug-challenged and control cysts incubated for 1 week on non-nutrient agar *E. coli* plates. Cysts were drug-challenged and incubated as outlined in the legend of Figure 4. Twenty-five images covering the whole central region, and eight images of the peripheral regions were recorded by bright field microscopy per plate. Trophozoites and cysts were counted on images and normalized as cells per mm^2^ for the regions indicated. For the graph displaying the cyst populations seen in the central region, the numbers of cysts seen at time point 0 hours were subtracted from each sample to identify the new formed cysts. Data are presented as mean + SD obtained from four independent biological experiments. * *P* < 0.05 (1-way ANOVA with Dunett's post hoc test between control and drug-challenged groups).

To quantitatively compare the efficacies of the drug treatments, we counted the numbers of trophozoites and cysts that were observed in the central regions and the peripheries at 1 week post-treatment (Fig. 5). On plates inoculated with the CH, HD, DD, and MF-treated cysts, trophozoite and cyst numbers were comparable to the ones seen on plates inoculated with unchallenged cysts (control) were observed for both the central and the peripheral regions. Plates inoculated with PHMB, PD, NM, and PVPI-treated cysts suspensions displayed on none of the four biological replicates any trophozoites or freshly formed cysts in the central and peripheral regions (*P* < 0.05). Similar observations were made with lysoform-treated cysts, which served in this assay as negative control.

## Discussion

There is controversy in the literature regarding the effectivity of different drugs against *Acanthamoeba* isolates in vitro and in vivo*.* We aimed to analyze the effects of nine potential anti-amoebic agents on *Acanthamoeba castellanii* trophozoites and cysts in vitro, using an LDH assay, trypan blue staining, and a non-nutrient *E. coli* agar plate assay. With these methods, we planned to gain insight into potential specific treatments against the *Acanthamoeba castellanii* isolate 1BU.

PHMB and CH are biguanide, membrane-acting agents. The literature on their in vitro cysticidal effect is controversial. Some authors describe them as the most promising agents against different *Acanthamoeba* strains in vitro, particularly against the highly resistant cyst form of the organism.^29^^–^^31^ In contrast, Sunada et al. observed only a weak cysticidal effect of PHMB.^21^ Other reports have considered PHMB equivalent to or more effective than CH against trophozoites and cysts of different *Acanthamoeba* species, or that CH is more effective.^27^^,^^32^

Diamidine drugs act on nucleic acids. PD (Brolene) was considered to have a poor cysticidal effect^21^ and HD (Hexacyl) was reported to have higher amoebicidal activity than PD.^33^ The efficacy of PD was found to be lower than that of CH or MF against *Acanthamoeba.*^34^^,^^35^

Natamycin (5%) is known to inhibit fungal growth by binding to sterols and by impairment of membrane fusion via perturbation of ergosterol-dependent priming reactions that precede membrane fusion.^36^ A case report described complete healing of AK within 2 months using 2% natamycin.^37^ Sunada et al. reported an excellent cysticidal effect of 5% natamycin.^21^ None of the drugs used in this study contained benzalkonium chloride (BAK), except the Natamet eye drops, which were preserved with 0.02% BAK. Heaselgrave et al.^38^ showed that BAK is toxic to *Acanthamoeba.* The natamycin solution used in our experiments and in the clinical practice also includes a 0.02% BAK as a preservative. Therefore, in this case we cannot exclude an additional anti-amoebic effect of BAK in NM-challenged samples.

MF is an intracellular targeting agent that can denature essential cell proteins and disrupt the cell membrane. MF was used successfully in amoebic encephalitis,^39^ and in several in vitro experiments against *Acanthamoeba.*^22^^,^^35^^,^^40^

PVPI is a broad-spectrum microbicide that destroys microbial protein and DNA.^41^ At 1%, PVPI causes ridges to form in the outer cyst wall and separation of the inner cyst wall from the outer one^21^ and was effective against *Acanthamoeba* in vitro*.* ^42^^,^^43^

With the increasing resistance of microorganisms to antibiotics, photodynamic therapy (PDT) may be a different treatment option.^44^ Crosslinking as PDT alone seemed not to be effective enough in AK.^45^^–^^47^ PDT using Ce6 and red light seemed to be effective in the treatment of *Pseudomonas aeruginosa* keratitis.^25^^,^^48^ Obviously, both adequate photosensitizer uptake by the microorganism and oxygen availability are critical to treatment efficacy, and these are extremely difficult to attain in double-walled *Acanthamoeba* cysts.^26^^,^^49^

Concerning the overall effect of the drugs, in our experiments, PHMB, CH, HD, PD, DD, NM, MF, and chlorin e6-PDT increased LDH activity and trypan blue positivity significantly in trophozoites (*P* < 0.01) compared with controls. All tested drugs also increased the percentage of trypan blue-stained 1BU cysts significantly (*P* < 0.01) compared with controls. All clinically used concentrations of the drugs significantly increased the trypan blue positivity of trophozoites and cysts (*P* < 0.01). Concerning different drug concentrations, the effects of 0.005% PHMB (*P* = 0.59) and 0.005% CH (*P* = 0.10) on *trophozoites* were weaker compared with other concentrations, and the effect of 0.005% CH (*P* = 0.29), 0.00095%, or 0.0038% Ce6 with illumination (*P* = 0.14 and *P* = 0.06, respectively) on cysts was lower than other applied concentrations, using the trypan blue assay. These results prove the potential of the clinically used drug concentrations but the ineffectivity of Ce6-PDT against *Acanthamoeba* cysts.

LDH and trypan blue assays are based on different action modes. The cytosolic protein LDH is released by damaged cells and tissue and is a commonly used quantitative marker in injuries and disease. An LDH increase in cell culture supernatants is considered to correlate with the loss of viability of the cell population.

Trypan blue assay results depend on the cell membrane permeabilities of the cell types. It is based on the principle that viable cells usually produce cell membranes that exclude this type of dye. Our findings presented here and elsewere^23^ suggest that increased levels of LDH in the supernatants and uptake of trypan blue by drug-treated *A. castellani* 1BU cells do not necessarily correlate with cell death. We recently described that drugs such as PHMB increase the uptake of ethidium homodimer by 1BU cells, a fluorescent dye that is usually utilized to indicate the proportion of late apoptotic / necrotic cells in a cell population, whereas a large proportion of ethidium homodimer positive cells were also positive for the cell-permeant dye calcein, indicating that these cells retained their ability to enzymatically convert the non-fluorescent calcein precursor calcein-AM into the fluorescent calcein at the same time.^23^

PHMB interacts with membrane phospholipids, propamidine inhibits phospholipid synthesis. Both lead to membrane leakage. Based on our results, we assume that the double membrane of the cysts, at least the outer membrane, is more sensitive to PHMB than the trophozoite membrane.

From the trypan blue assay, we can draw a conclusion which drug has more impact on the cell membrane, meanwhile from the LDH assay, we can draw the conclusion of which drug induces more human tissue damage, two types of information that are both of importance in our opinion. However, based on our findings made with the non-nutrient agar *E. coli* plate assay, we conclude that neither the LDH release assay nor the trypan blue assay provides reliable information on the killing capacity of the anti-amoebic drugs tested here.

*Acanthamoeba* cysts have a double cellulose wall, which is extremely resistant to environmental damage. By utilizing the trypan blue assay, we observed that all drugs significantly increased the uptake of the dye by *A.* castellani 1BU trophozoites and cysts, suggesting that all drugs are cytotoxic for both morphotypes. To corroborate this hypothesis, we also determined the drug efficacies with a culture-based viability assay, the non-nutrient agar *E.* *coli* plate assay, in which *E. coli* cells serve as nutrition for the *Acanthamoeba*. This set of experiments revealed a number of striking observations: cysts treated with the drugs CH, HD, DD, and MF retained their ability to excyst and formed normal-shaped trophozoites on the non-nutrient agar *E. coli* plates that were able to migrate into the peripheral regions of the plates and to form new cysts (Fig. 4). Of note, after 1 week of incubation, cysts treated with the above mentioned drugs produced a comparable number of trophozoites and new cysts on the *E. coli* decorated agar surface than the untreated cysts, irrespective of the large differences in trypan blue staining rates observed between the drug-treated groups and the control.

Cysts challenged with the drugs PHMB, PD, NM, and PVPI, on the other hand, failed to excyst, and no trophozoites were observed on the *E. coli* decorated agar surface even after 3 weeks of incubation. These findings demonstrate that the latter drugs are capable of suppressing the encystment of 1BU cysts and suggest that these drugs might be able to kill the cysts, at least under in vitro conditions. However, to be effective in vivo, drugs need to accumulate in sufficient amounts on / in the *Acanthamoeba* trophozoites / cysts in the presence of host factors / tissue, which might sequester the drug from its target, and potential cytotoxic effects of the anti-amoebic agents on human corneal cells also have to be considered, in case of clinical use.^50^

It should be also noticed that, in our study, only one *Acanthamoeba* isolate was analyzed, and it cannot be excluded that other *Acanthamoeba* genotypes may respond to the tested drugs in a different way.^51^ Experiments are ongoing in our laboratories to address this issue.

In conclusion, our data demonstrate that the off-label AK drugs PHMB, PD, NM, and PVPI are, under in vitro conditions, more effective against *Acanthamoeba castellanii* strain 1BU than CH, HD, DD, and MF. Our findings also suggest that the non-nutrient agar *E.* *coli* plate assay should be considered as method of choice for the in vitro analysis of the treatment efficacy of anti-amoebic agents.
